# Supplementary figures and images for: Seasonal and predator-prey effects on circadian activity of free-ranging mammals revealed by camera traps
Source: PeerJ. 2018 Nov 21;6:e5827. doi: 10.7717/peerj.5827 (PMC6252065; doi:10.7717/peerj.5827)

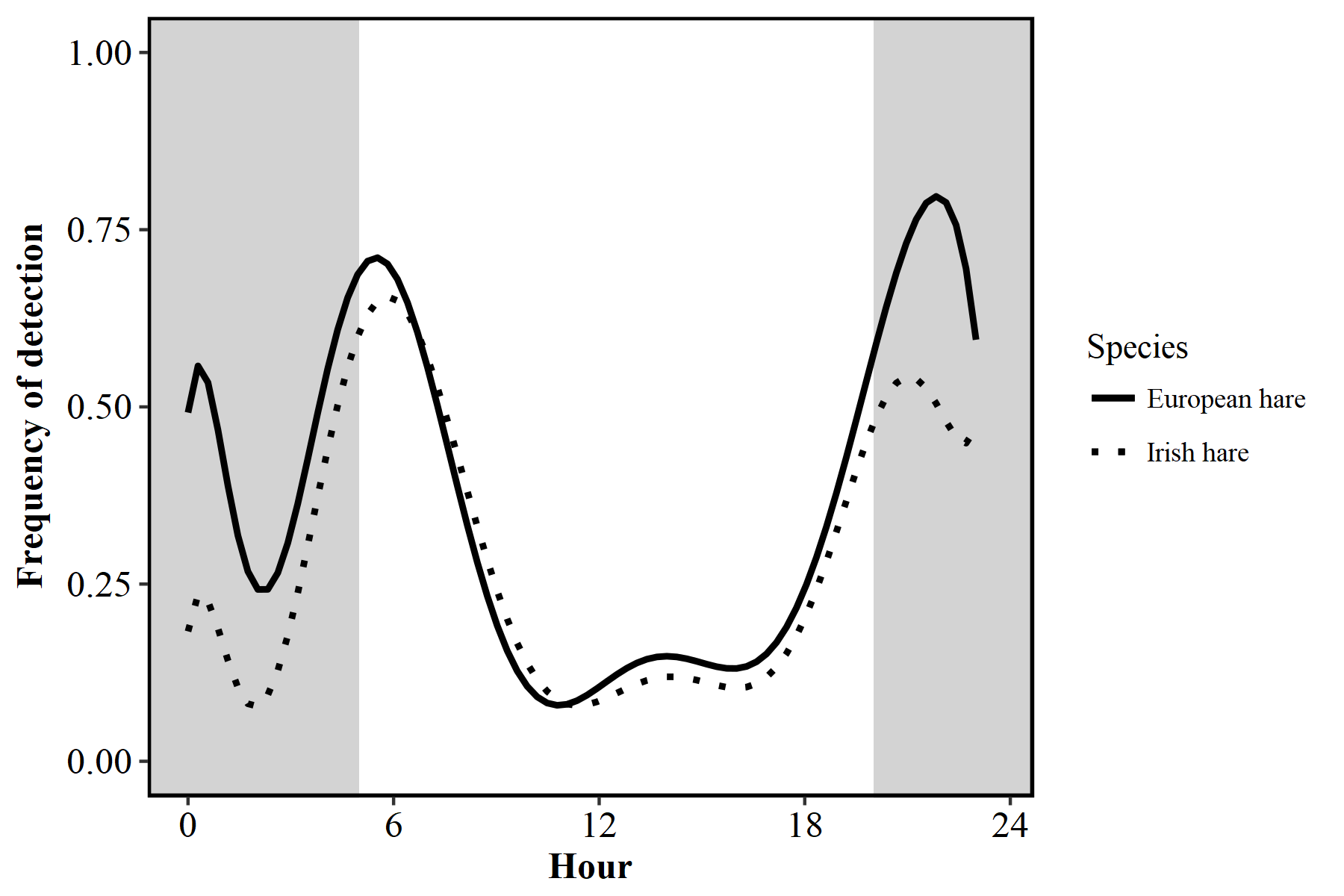

Supplement: Supplemental Information 1 — Due to the close similarities in activity patterns, species were grouped as ‘hares’ for the purposes of this study. [file peerj-06-5827-s001.png]

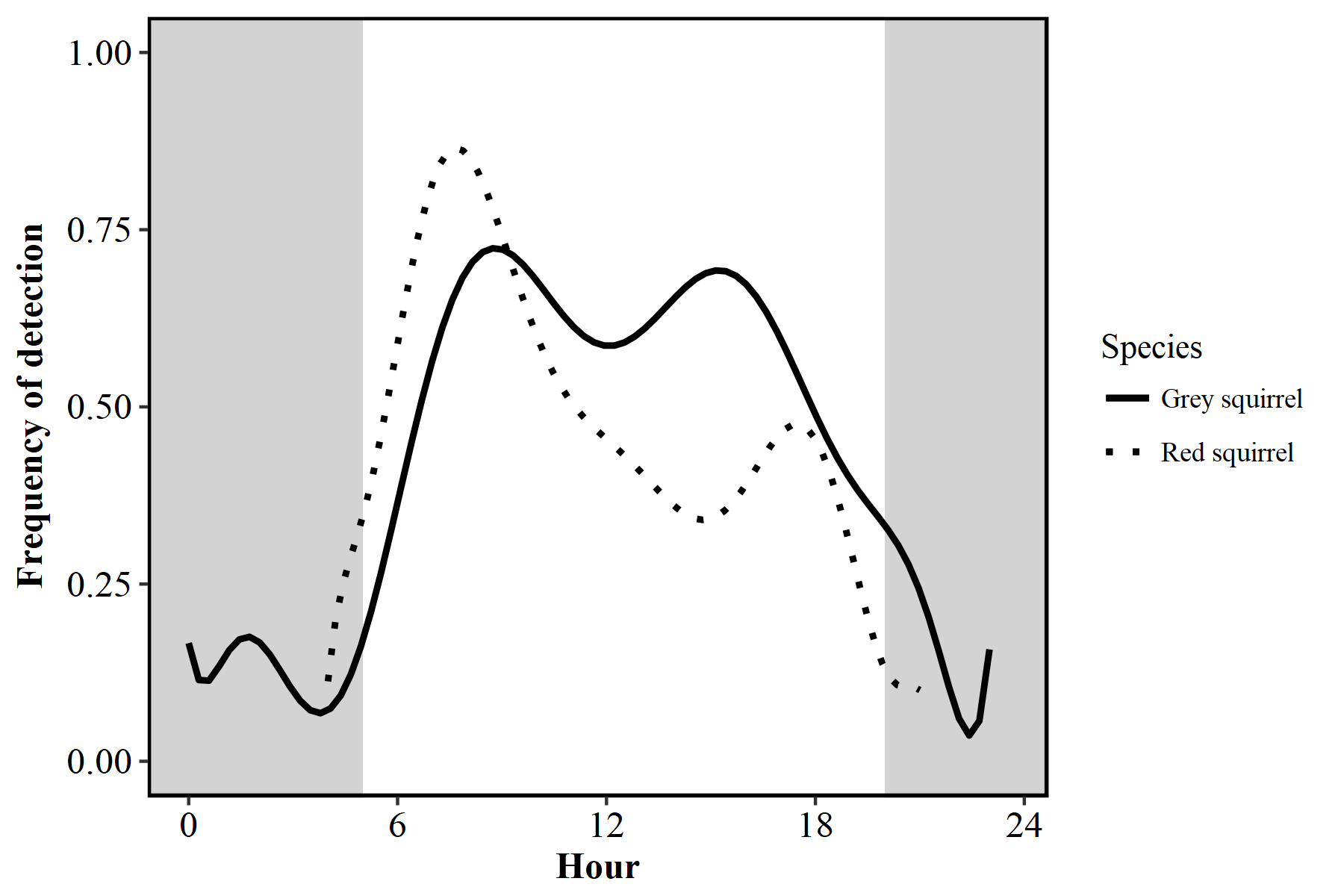

Supplement: Supplemental Information 2 — Due to the close similarities in activity patterns, species were grouped as ‘squirrels’ for the purposes of this study. [file peerj-06-5827-s002.png]

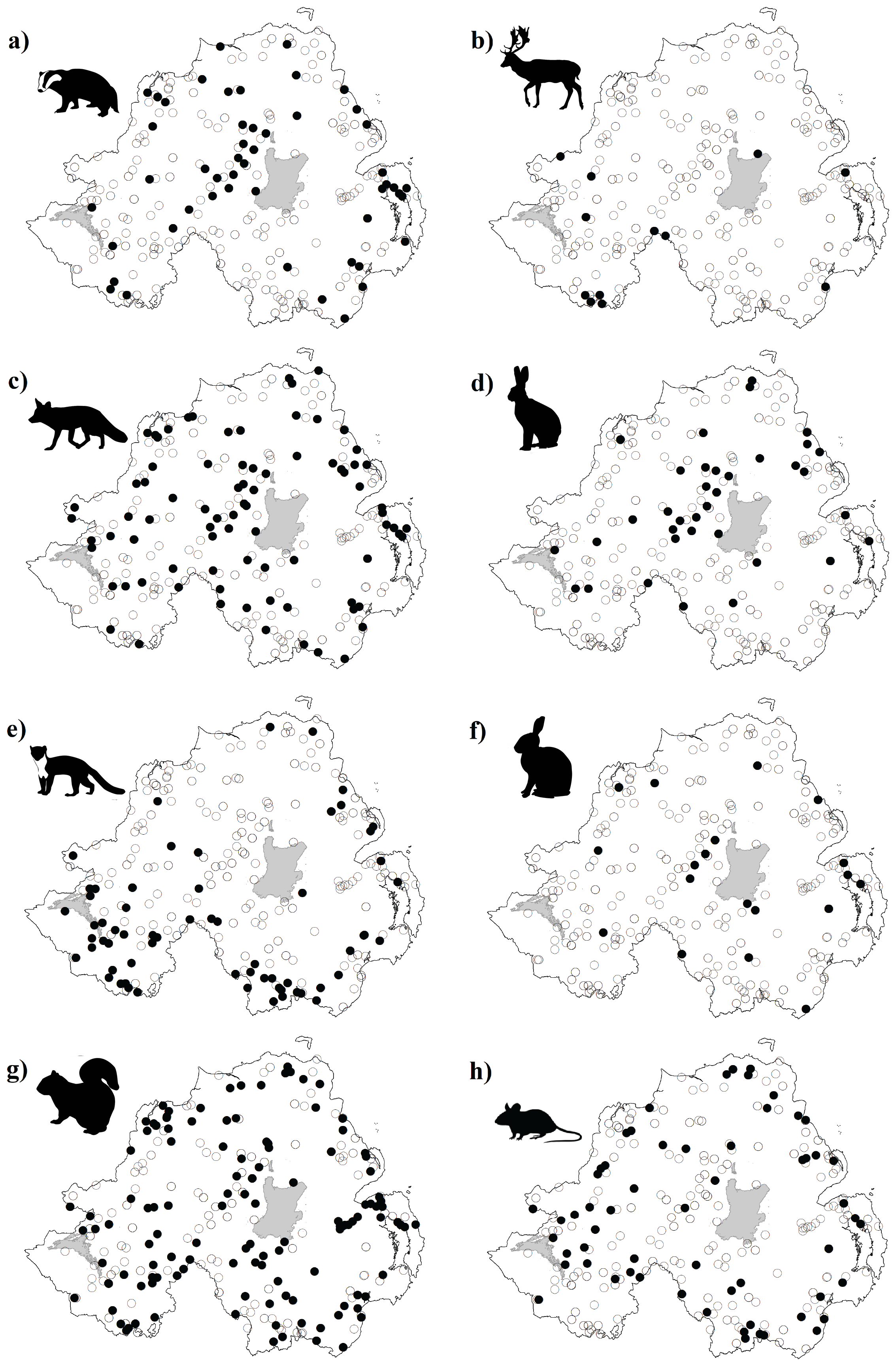

Supplement: Supplemental Information 3 — (a) Badger (Meles meles), (b) fallow deer (Dama dama), (c) fox (Vulpes vulpes), (d) hare (Irish hare, Lepus timidus hibernicus and European hare, L. europaeus), (e) pine marten (Martes martes), (f) rabbit (Oryctolagus cuniculus), (g) squirrel (grey squirrel, Sciurus carolinensis and red squirrel, S. vulgaris), and (h) wood mouse (Apodemus sylvaticus). • = species detected at that location; ○ = species not detected at that location. [file peerj-06-5827-s003.png]

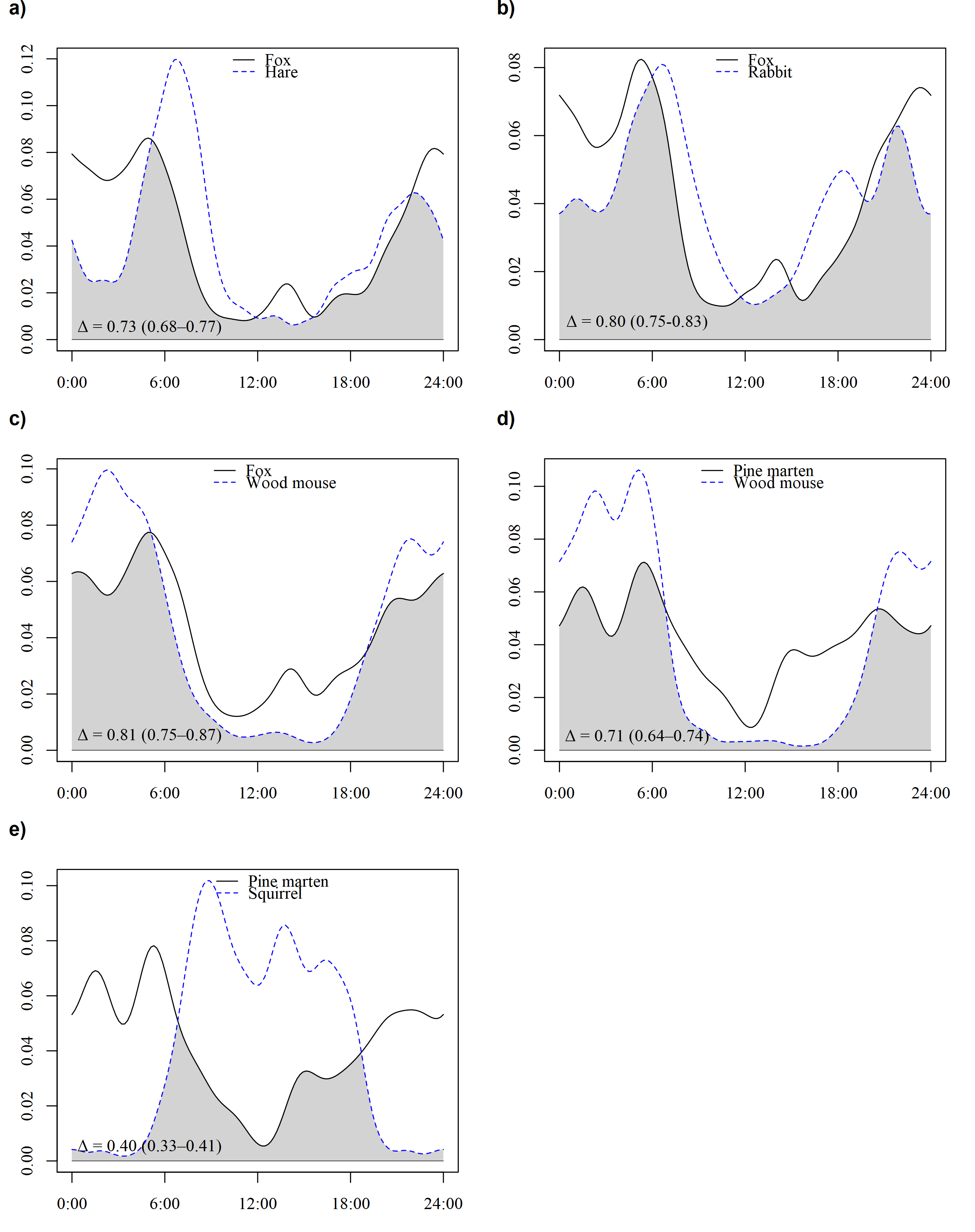

Supplement: Supplemental Information 4 — The overlap coefficient (Δ) ± 95% Confidence Intervals (CIs) are also given. [file peerj-06-5827-s004.png]
